# Supplementary material for: Iterative improvement in the automatic modular design of robot swarms
Source: PeerJ Comput Sci. 2020 Dec 7;6:e322. doi: 10.7717/peerj-cs.322 (PMC7924708; doi:10.7717/peerj-cs.322)
Supplement: Supplemental Information 3 [file peerj-cs-06-322-s003.zip › argos3/doc/api/standalone/a00461_source.html]

ARGoS: plugins/robots/foot-bot/simulator/footbot\_distance\_scanner\_equipped\_entity.h Source File


- Main Page
- Related Pages
- Namespaces
- Classes
- Files

- File List
- File Members

# plugins/robots/foot-bot/simulator/footbot\_distance\_scanner\_equipped\_entity.h

Go to the documentation of this file.

```
00001 
00007 #ifndef FOOTBOT_DISTANCE_SCANNER_EQUIPPED_ENTITY_H
00008 #define FOOTBOT_DISTANCE_SCANNER_EQUIPPED_ENTITY_H
00009 
00010 namespace argos {
00011    class CFootBotDistanceScannerEquippedEntity;
00012 }
00013 
00014 #include <argos3/core/simulator/entity/entity.h>
00015 #include <argos3/core/utility/math/vector3.h>
00016 
00017 namespace argos {
00018 
00019    class CFootBotDistanceScannerEquippedEntity : public CEntity {
00020 
00021    public:
00022 
00023       ENABLE_VTABLE();
00024 
00025       enum EMode {
00026          MODE_OFF,
00027          MODE_POSITION_CONTROL,
00028          MODE_SPEED_CONTROL
00029       };
00030 
00031    public:
00032 
00033       CFootBotDistanceScannerEquippedEntity(CComposableEntity* pc_parent);
00034 
00035       CFootBotDistanceScannerEquippedEntity(CComposableEntity* pc_parent,
00036                                             const std::string& str_id);
00037 
00038       virtual void Reset();
00039 
00040       virtual void Update();
00041 
00042       inline UInt32 GetMode() const {
00043          return m_unMode;
00044       }
00045 
00046       inline void SetMode(UInt32 un_mode) {
00047          m_unMode = un_mode;
00048       }
00049 
00050       inline const CRadians& GetRotation() const {
00051          return m_cRotation;
00052       }
00053 
00054       void SetRotation(const CRadians& c_rotation);
00055 
00056       inline Real GetRotationSpeed() const {
00057          return m_fRotationSpeed;
00058       }
00059 
00060       inline void SetRotationSpeed(Real f_speed) {
00061          m_fRotationSpeed = f_speed;
00062       }
00063 
00064       virtual std::string GetTypeDescription() const {
00065          return "distance_scanner";
00066       }
00067 
00068    private:
00069 
00070       UInt32 m_unMode;
00071       CRadians m_cRotation;
00072       Real m_fRotationSpeed;
00073 
00074    };
00075 }
00076 
00077 #endif
```

---

Generated on 10 Jul 2018 for ARGoS by 
 1.6.1
